# Stress and Tinnitus; Transcutaneous Auricular Vagal Nerve Stimulation Attenuates Tinnitus-Triggered Stress Reaction

**DOI:** 10.3389/fpsyg.2020.570196

**Published:** 2020-09-17

**Authors:** Jukka Ylikoski, Marika Markkanen, Ulla Pirvola, Jarmo Antero Lehtimäki, Matti Ylikoski, Zou Jing, Saku T. Sinkkonen, Antti Mäkitie

**Affiliations:** ^1^Helsinki Ear Institute, Helsinki, Finland; ^2^Salustim Group Inc., Kempele, Finland; ^3^Department of Otolaryngology-Head & Neck Surgery, University of Helsinki and Helsinki University Hospital, Helsinki, Finland; ^4^Department of Anatomy, University of Helsinki, Helsinki, Finland; ^5^Molecular and Integrative Biosciences Research Program, University of Helsinki, Helsinki, Finland; ^6^Department of Otolaryngology-Head and Neck Surgery, Center for Otolaryngology-Head & Neck Surgery of Chinese PLA, Changhai Hospital, Second Military Medical University, Shanghai, China

**Keywords:** stress, tinnitus, patients, parasympathetic, vagus, neuromodulation

## Abstract

**Introduction:**

Tinnitus can become a strong stressor for some individuals, leading to imbalance of the autonomous nervous system with reduction of parasympathetic activity. It can manifest itself as sleep disturbances, anxiety and even depression. This condition can be reversed by bioelectrical vagal nerve stimulation (VNS). Conventional invasive VNS is an approved treatment for epilepsy and depression. Transcutaneous VNS (taVNS) stimulating the auricular branch of the vagus nerve has been shown to activate the vagal pathways similarly as an implanted VNS. Therefore, taVNS might also be a therapeutic alternative in health conditions such as tinnitus-related mental stress (TRMS). This retrospective study in 171 TRMS patients reports the clinical features, psychophysiological characteristics, and results of the heart rate variability (HRV) tests before and after test-taVNS. This study also reports the therapy outcomes of 113 TRMS patients treated with taVNS, in combination with standard tinnitus therapy.

**Methods:**

Diagnostic tinnitus and hearing profiles were defined. To detect possible cardiac adverse effects, test-taVNS with heart rate monitoring as well as pre- and post-stimulation HRV tests were performed. Daily taVNS home therapy was prescribed thereafter. To assess therapeutic usefulness of taVNS, 1-year follow-up outcome was studied. Results of HRV tests were retrospectively analyzed and correlated to diagnostic data.

**Results:**

The large majority of patients with TRMS suffer from associated symptoms such as sleep disturbances and anxiety. Baseline HRV data showed that more than three quarters of the 171 patients had increased sympathetic activity before test-taVNS. Test-taVNS shifted mean values of different HRV parameters toward increased parasympathetic activity in about 80% of patients. Test-taVNS did not cause any cardiac or other side effects. No significant adverse effects were reported in follow-up questionnaires.

**Conclusion:**

TRMS is an example of a stress condition in which patients may benefit from taVNS. As revealed by HRV, test-taVNS improved parasympathetic function, most efficiently in patients with a low starting HRV level. Our tinnitus treatment program, including taVNS, effectively alleviated tinnitus stress and handicap. For wider clinical use, there is a great need for more knowledge about the optimal methodology and parameters of taVNS.

## Introduction

All our unconscious bodily functions are controlled by the autonomic nervous system (ANS), particularly by the CAN (Benarroch, 1993). The most common cause for the dysfunction of CAN is stress, the major cause of deteriorating health conditions and illnesses. CAN initially reacts to stressor effects with sympathetic fight/flight response that is restored back to normal by the parasympathetic nervous systemś relax/digest response (Selye, 1950). Many illnesses result from the inability of the parasympathetic activity to restore the ANS balance (for review see McEwen, 2000; McEwen and Akil, 2020). These two circuits, sympathetic and parasympathetic systems, are constantly interacting. This interaction is reflected by HRV that, hence, is a read out of ANS balance. HRV may consequently serve as a measure of stress (Akselrod et al., 1981; Thayer et al., 2012). As the vagal system with the vagus nerve in front is responsible for parasympathetic activity, neuromodulation via VNS can serve as targeted treatment in stressful conditions. VNS has been conventionally performed for more than two decades to treat severe epilepsy and depression by applying an electrode surgically implanted to the cervical trunk of the vagus nerve. More recently, it has been shown by electrophysiological and neuroimaging studies that taVNS of the ABVN activates central vagal pathways similarly as VNS with an implanted electrode (Kraus et al., 2007; Dietrich et al., 2008; Frangos et al., 2015; Yakunina et al., 2017; Badran et al., 2018a).

ANS imbalance is most often a result of the individual’s exposure to concurrent stressors. Therefore, the stress-triggered clinical picture is often very heterogeneous. On the contrary, tinnitus (ringing in the ears) as a stressor usually results in a relatively regular SR in otherwise healthy individuals. Therefore, individuals with TRMS seem to be an optimal target group for investigations of the effects of taVNS on stress in patients.

Tinnitus is considered to be generated in the auditory periphery (cochlea-cochlear nerve), detected in the subcortical centers according to the lines of pattern recognition principles, and perceived and evaluated in the auditory cortex with significant participation of the limbic system and prefrontal and other cortical areas (Jastreboff, 1990). Tinnitus sound itself usually constitutes only minor symptoms. However, tinnitus connected with fear (that it is maintained or even growing worse) and threat (that it is a sign of a serious illness) leads to automatic negative thoughts, developing a SR (arousal) that may potentiate sleep problems, anxiety and depression. The end result all of this is a vicious cycle, SR worsening tinnitus and increased tinnitus worsening stress.

This was taken into consideration in the neurophysiological tinnitus model by Jastreboff (1990) and it formed the basis for the development of the TRT program (Jastreboff and Hazell, 1993). The target of TRT therapy is the stress-arousal caused by tinnitus and it leads to distress that prevents habituation. The goal of TRT is to remove the negative perception of tinnitus from patient’s consciousness, thereby facilitating habituation. Furthermore, the rationale of TRT is to attenuate the conditioned stress-response (arousal) with associated sympathovagal imbalance by stimulating the parasympathetic system (Jastreboff and Hazell, 1993). TRT is a program consisting of diagnostics, instructive counseling and sound therapy, each acting in concert with the aim to stimulate the parasympathetic system.

Plans for this study were started after Engineer et al. (2011) reported that maladaptive neuronal plasticity of the central auditory system, thought to be behind tinnitus in an animal tinnitus model, can be reversed through (invasive) VNS. It was clear that conventional (invasive) VNS would not be the optimal treatment for patients with tinnitus. Therefore, we first had to develop a method and device for noninvasive VNS (taVNS). When the device development was completed, we had to start to test-use it particularly for cardiac safety.

This retrospective study reports the clinical, audiological and psychophysical diagnostic results in a historical group of 171 patients managed for TMRS. The study also reports the results of the 1-year follow-up outcome study with patients treated with taVNS. Because stress levels of patients needed to be measured and because of the novelty of taVNS and the potential cardiac complications reported by VNS, the HRV test and test-taVNS with HR monitoring were considered obligatory. We performed HRV test both before and after test-taVNS. Our data show that taVNS is safe and improves parasympathetic activity and, in conjunction with TRT, attenuates tinnitus severity based on results of symptom questionnaires.

## Patients and Methods

The present series consists of 171 consecutive patients (67 female, 104 male, mean age 49 years, range 17–84) who visited the Tinnitus Clinic of Helsinki Ear Institute between November 2014 and December 2017 due to annoying tinnitus. We have developed our own modification of TRT that is named TCPT. Its main constituents include diagnostic profiling of tinnitus and hearing, counseling, sound therapy, and a sleep module. In order to strengthen parasympathetic activation, taVNS was added to the program in 2014. In addition to other diagnostics, HRV tests and a 15–60 min test-taVNS were performed and, as adjunctive to other TCPT therapy, a taVNS device was prescribed as home-therapy if tinnitus had been defined as moderate or severe (THI, questionnaire score higher than 34/100). Some patients with lower THI scores were also instructed for taVNS treatment if they showed special interest in the device or complained of being particularly stressed. At the initial office visit, extensive otological and audiological examinations with profiling of tinnitus and hearing were done using different structured diagnostic forms and questionnaires. For clinical evaluation of the subjective severity of tinnitus annoyance and associated symptoms such as sleep disturbance and anxiety levels were quantified based on VAS questions.

Each patient’s stress level was evaluated by HRV testing. The patient was then (for safety reason) test-stimulated with the Salustim taVNS device (Helsinki Ear Institute) continuously for 15–60 min after which a new HRV test was performed. We have earlier shown by magnetoencephalography that the amplitudes of auditory N1m responses in the auditory cortex are reduced by using this taVNS method (Lehtimäki et al., 2013). As there were no adverse effects related to the test-taVNS, all tested patients were then instructed to use the taVNS device at home 60–90 min per day. The long-term therapeutic outcomes of our first 113 patients were studied with structured questionnaires about 1 year after the first visit. Such follow-up data was possible to obtain during office visits scheduled according to the TCPT program or by telephone interviews in 78 patients (69%) (54 males and 59 females; age range from 18 to 84 years).

### HRV Test and Heart Rate Monitoring During Test-taVNS

The main aim of HR monitoring was to detect possible cardiac side-effects during the first 15–60 min of taVNS stimulation. Other reasons were to measure the mental stress level of patients and to collect HRV data for later analyses to study whether HRV could be used for selection of presumably taVNS-responding patients. In this study, we report the results of HRV analyses and specifically correlate HRV results to clinical data in a total of 171 patients. We have previously described in detail our HRV testing procedure (Ylikoski et al., 2017). Briefly, for analyzing the dynamics of HRV signals (R-R intervals), the eMotion HRV measurement system (Mega Electronics Ltd., Kuopio, Finland) was used. Stress test with a HRV scan was performed with the patient breathing with a parasympathetic stimulating respiratory rate during which the R-R interval variability was registered by wrist electrodes with a one-lead ECG. In the eMotion HRV measurement, artifacts and interruptions were eliminated with high-end technology (and disposable surface electrodes) (Malik, 1996; Tarvainen et al., 2009). Only tests with 100% measurement quality in ECG monitoring were used for evaluation. The following HRV parameters were measured during the 1-min deep-breathing test (one-min DBT): mean HR, amplitude and ratio of HR oscillation (E-I difference, E/I ratio) (E-I = difference between the highest and the lowest HR within a breathing cycle), RMSSD (root mean square of the successive differences), SDNN (standard deviation of the R-R intervals), and Power LF (LF = low frequency; 0.04–0.15 Hz). One-min DBT was followed by 5-min short-term HRV (s-HRV) where the HRV parameters HR, SD1 (width of the Poincare plot, reflecting short-term variability), SD2 (length of the Poincare plot, reflecting short-term variability), SDNN, Stress Index, Power HF (HF = high frequency; 0.15–0.4 Hz), Power LF, Power VLF (very low frequency), and Total Power were determined as well. Parameters were compared through correlation analysis and agreement analysis by Bland-Altman plots. The results of HRV tests were evaluated on the basis of eMotion tests performed in normal individuals (Camm et al., 1996; Tarvainen et al., 2009; Weinschenk et al., 2016).

### Vagal Somatosensory Evoked Potential Test

In order to biomonitor the electrical stimulation of ABVN, we measured the VSEP response (Fallgatter et al., 2003). We used EGI GTEN 100 EEG system with 8 kHz collection rate (256 electrodes) (Palva and Palva, 2018) and with different stimulation parameters. We used uni- or bipolar pulses, pulse width 100–560 microseconds, frequencies 1, 2, 4, 8, 15, 20, 25, 30 Hz, amplitude at or just below the pain threshold, and delivered 200–500 epochs at each setting.

### taVNS

We used the taVNS instrument consisting of one ear clip electrode connected to a wired TENS-neurostimulating device (Salustim, Helsinki Ear Institute). The clip-electrode was placed on the tragus of the left ear. The clinical efficacy of taVNS requires activation of the thick myelinated afferent fibers of the vagus nerve. Fibers of a sensory peripheral nerve, such as the ABVN, mediate touch sensation. Consequently, the stimulus intensity of taVNS will be adjusted to a level above the individual’s detection threshold and clearly below the individual’s pain threshold. The taVNS device offers a stimulus intensity between 0.1 and 30 mA with a stimulation frequency of 25 Hz and pulse duration of 250 μs. After the individual adjustments the level of stimulation in patients ranged from 0.3 to 3.0 mA. The 15–60 min continuous test stimulation was performed under medical supervision in the office, with continuous HR monitoring. After the initial test stimulation, patients were instructed to use the taVNS device at home for 60–90 min daily, 5 days a week.

### Statistical Analyses

Statistical analyses were performed using GraphPad Prism 8 (GraphPad Software, La Jolla, CA, United States). Normality of data was tested with D’Agostino & Pearson test. The non-parametric Wilcoxon’s matched pairs signed rank test was used to estimate the *p*-values between pre/post -taVNS data, as all data did not follow a normal distribution. Comparison between age groups was also done using the non-parametric Kolmogorov–Smirnov test. Comparison between super-responders vs. non -responders was done using unpaired two-tailed Student’s *t*-test as the data followed a normal distribution. Data is presented as mean ± standard deviation (SD). Statistical significance was set up at *P* < 0.05. Bonferroni adjustment were performed for multiple comparisons by dividing the initial significance level of 0.05 by the number of tests to obtain a modified significance level. Treatment effect sizes were calculated by Cohen’s *d* as the difference in means, divided by the pooled standard deviation of the two means (Cohen’s, 1988). The magnitude of Cohen’s *d* can be expressed as small (0.2), moderate (0.5), and large (0.8).

## Results

### Baseline Values

**FIGURE 1:**
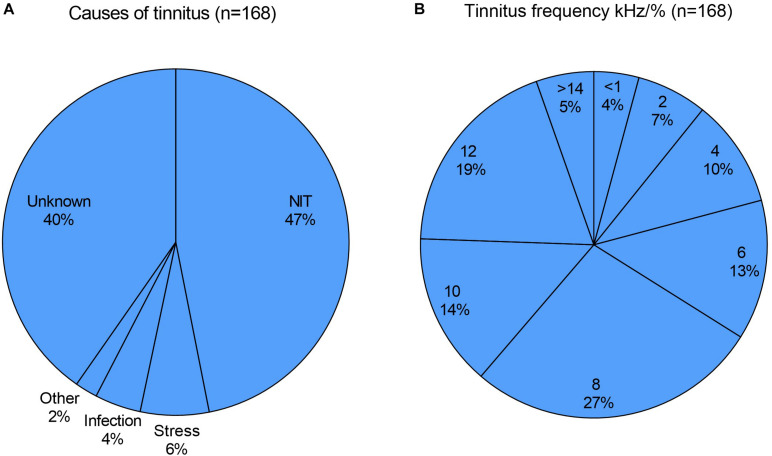
**(A)** The most common cause of tinnitus was acoustic overstimulation (noise-induced tinnitus, NIT; 47%), followed by self-reported stress (6%), otitis media (4%) and other causes (2%). The cause was unknown in 40%. **(B)** The most common tinnitus frequency was between 7 and 9 kHz (27%), (marked as 8), followed by 11–14 kHz (19%), (marked as 12), 9–11 kHz (14%), (marked as 10), 5–7 kHz (13%), (marked as 6). In 9 patients (5%) tinnitus frequency was >14.0 kHz, in 17 patients (10 %) between 3 and 5 kHz (marked as 4), in 11 patients (7 %) between 1 and 3 kHz (marked as 2) and in seven patients (4%) < 1.0 kHz.

All the patients had clinically relevant tinnitus; chronic in two thirds, subchronic in about 20% and acute (tinnitus duration less than 3 months) in about 15%. In acute cases, most patients had visited our tinnitus clinic one to 6 weeks after the start or worsening of tinnitus. The most common cause of tinnitus was acoustic overstimulation (“NIT”, 47%), usually of a result of exposure to loud music in a festival or restaurant. About one third of NIT cases was defined as music-induced tinnitus (MIT). The MIT patients were usually professional or hobby musicians. Other causes were self-reported stress (6%), otitis media (4%) and other causes such as flight travel (2%). The cause of tinnitus was unknown in 40% of cases (Figure 1A).

Otological examinations were normal and there were no audiometrically detectable acute hearing impairment, except in five patients that had been exposed to shooting noise. They had a mild (10–25 dB) dip-type hearing loss either at 4 or 6 kHz. Two thirds of patients showed normal or age-related hearing loss in pure tone audiometry. Tinnitus was high-pitched (8 kHz or higher) in two thirds (66%) and >4 kHz in about 85% of cases. The most common tinnitus frequency was detected between 7 and 9 kHz (27%), followed by 11–14 kHz (19%), 9–11 kHz (14%) and 5–7 kHz (13%) (Figure 1B).

**FIGURE 2:**
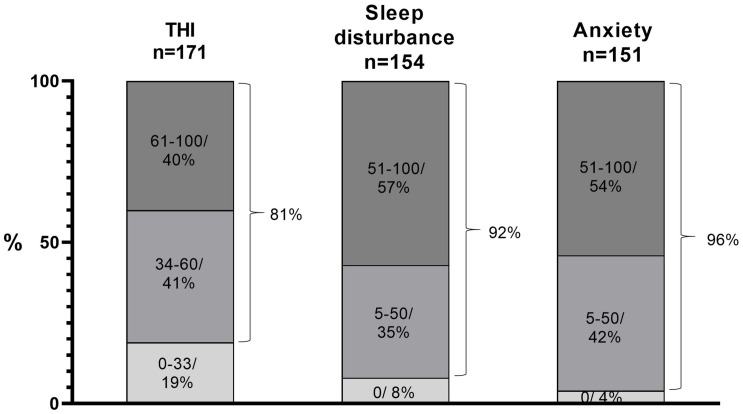
THI was between 34 and 100 in 81% of patients. The mean THI value was 55 and the mean VAS-scaled (0–100) values of both loudness and annoyance were 55. Tinnitus was associated with sleep disturbances in 92% and with anxiety in 96%, both being severe or very severe (>50/100 in VAS scale) in 57% and 54% of patients, respectively.

The mean THI was 55, and it was between 34 and 100 in 81% of patients indicating moderate or severe tinnitus (Figure 2). Tinnitus was frequently associated with sleep disturbances (92%) and anxiety (96%) (Figure 2). Both sleep disturbances and anxiety were severe or very severe (above 50/100 in VAS in more than half of the patients (57% and 54%, respectively). It may be noteworthy that the patients were typically stressed because, prior to visiting us, they had visited general practitioners or ENT specialists and had received negative counseling (“nothing can be done”, “you just have to learn to live with it”). One third of patients had a history or ongoing therapy of depression, ranging from mild (12%) to moderate (18%) to severe (3%).

### HRV Tests and Results of Test-taVNS

The main aim of the initial test-taVNS was to ascertain the cardiac safety of the method. We found no cardiac adverse effects in our 171 patients. This applies actually to more than 250 taVNS patients treated so far by us: none of them reported cardiac or other serious side-effects during the first test-taVNS or during home treatment. Several of our patients have used the taVNS device regularly, practically daily for 2–3 years, some up to 5 years.

Baseline data from 1-min DBT-HRV and 5-min short-term HRV (s-HRV) showed that more than three quarters of TRMS patients had increased sympathetic activity before test-taVNS. This was deduced from the stress index (data not shown) and the HRV age, which was in this patient population approximately 16 years higher than the mean chronological age. Table 1 shows the mean values of different HRV parameters, considered to describe the vasovagal tone and the changes of HRV parameters immediately after the 15–60 min test-taVNS. The taVNS significantly increased all HRV parameters: the mean R-R interval in 81%, Flexibility = E-I (difference between the highest and the lowest heart rate within a breathing cycle) in 63%, Dynamics = RMSSD, (root of the mean square of successive differences) in 69%, Tone = mean HR in DBT in 68% of patients, and decreased the HRV age by 9 years (Table 1).

**TABLE 1:** Mean HRV test data of 171 patients with TRMS.

	R-R-interval	HRV-age	Flexibility %	Dynamic % (RMSSD)	Tone %
	**pre/post taVNS**	**pre/post taVNS/chronol**	**pre/post taVNS**	**pre/post taVNS**	**pre/post taVNS**

Mean	815/868	65/56/49	32/44	32/49	38/49
SD	141/140	22/23/16	28/30	30/34	30/30
*p*-Value	<0,0001	<0,0001	<0,0001	<0,0001	<0,0001
Cohen’s *d*	0,377	0,400	0,414	0,530	0,367
taVNS induced change	+52 (6.4%)	−9 (13.7%)	+11 (34.5%)	+18 (53.2%)	+12 (30.5%)
Increased	139 (81.3%)	12 (7.0%)	107 (62.6%)	118 (69.0%)	116 (67.8%)
Decreased	32 (18.7%)	135 (79.0%)	36 (21.1%)	16 (9.4%)	22 (12.9%)
Unhanged	–	24 (14.0%)	28 (16.4%)	37 (21.6%)	33 (19.3)

*The taVNS significantly increased all HRV parameters: the mean R-R interval in 81%, Flexibility in 63%, Dynamics (RMSSD) in 69%, Tone in 68% of patients, and decreased the HRV age by 9 years. The mean pre-taVNS HRV age was 16 years higher than the chronological age. The taVNS induced change represents the mean difference between pre- and post-taVNS values of all patients. The mean percentage change is also shown. Increases of RR-interval, flexibility, RMSSD and tone and decrease of HRV-age indicate increased parasympathetic or decreased sympathetic tone. Pre = baseline data; post = post-taVNS stimulation data. Wilcoxon’s matched pairs signed rank test was used to calculate *p*-values. The Bonferroni adjustment method for multiple testing produced a rejection *p*-value of 0.01. All *p*-values remained statistically significant. Effect sizes (Cohen’s *d*) ranged from small to medium, the largest was observed in Dynamic % (RMSSD).*

From our previous experience (Ylikoski et al., 2017) and from the results of the current study, we deduced that R-R interval, HRV age and RMSSD are the most useful markers for stimulation changes in HRV (Table 1). RMSSD is mainly related to beat-to-beat variations reflecting parasympathetic output (Faber et al., 1996). In practice, the change in vasovagal tone is best illustrated by the HRV age that was determined by algorithms based on values of HRV parameter values, as described by Weinschenk et al. (2016).

If in cases where R-R interval was decreased (in 19%) after test-taVNS, the results of HRV age were taken into consideration, either the HRV age or R-R interval showed increased parasympathetic activity in more than 95% of cases.

**FIGURE 3:**
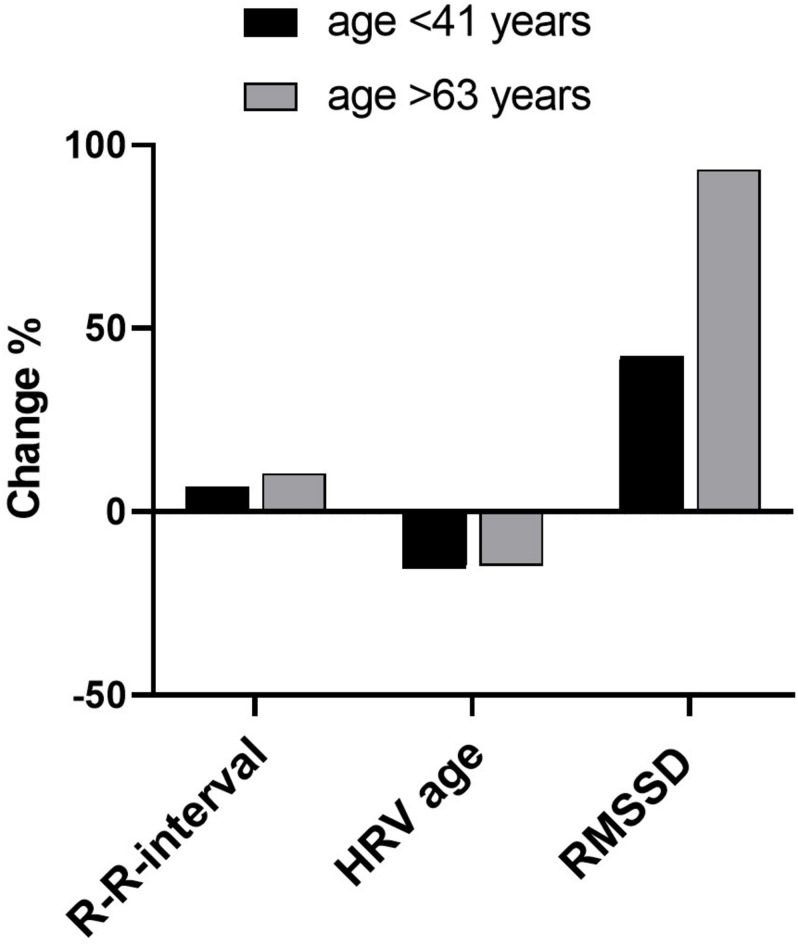
Correlations between HRV parameters between Group A (below 41 years) and Group B (above 63 years) shown in Table 2. The taVNS-induced numerical increases of these three HRV parameters were greater in the older group. However, only the RMSSD changes reached statistical significance.

To compare the changes in R-R interval, RMSSD and HRV age with the age of patients, we selected the youngest patients (age < 41 years) for one group (*n* = 41) and the oldest patients (age > 63 years) for another group (*n* = 21) (Table 2, Figure 3). Test-taVNS increased all the three HRV parameters much more often in patients of the older group: R-R interval in 86% in >63 group, 73% in < 41 group, RMSSD in 81% in >63 group, 66% in < 41 group and HRV age in 81% in >63 group, 58% in < 41 group (Table 2). The taVNS-induced numerical increases of the three HRV parameters were also greater in the older patients. However, only the RMSSD changes reached statistical significance (Table 2, Figure 3). This indicates that taVNS is more efficient in older patients, a result that could be explained by lower starting levels of HRV in this group due to age-related decline in parasympathetic activity. The magnitude of taVNS-induced changes has been shown to be higher in individuals with lower starting HRV level (Bretherton et al., 2019).

**TABLE 2:** Correlations between HRV parameters and scores of sleep and anxiety between age groups.

	R-R-interval	HRV-age	RMSSD	sleep/anxiety
	pre/post taVNS	pre/post taVNS	pre/post taVNS	
All patients (*n* = 171)	+52 (6.4%)	−9 (13.7%)	+18 (53.2%)	55
<41 year, *n* = 41	+53,6 (6,9%)	−7,76 (−15,5%)	+15,2 (42,6%)	61
>63 year, *n* = 21	+85,0 (10,5%)	−11,7 (−14,7%)	+30,1 (93,3%)	52
*p*-Values	0,515	0,0718	0,0392	0,456
Cohen’s *d*	0,395	0,315	0,574	0,276

*Correlations between HRV parameters (R-R interval, HRV age and RMSSD), and scores of questionnaires for sleep and anxiety between Group A (age < 41 years) and Group B (age > 63 years). Test-taVNS increased all three HRV parameters much more often in patients of the older group: R-R interval in 86% in >63, 73% in < 41 groups, RMSSD 81% in >63, 66% in < 41 groups and HRV age 81% in >63, 58% in < 41 group. Shown is the mean taVNS induced change (mean difference between pre- and post-taVNS values) of all patients as well as of the two age groups. The taVNS-induced numerical increases of these three HRV parameters were also greater in the older group. Only the RMSSD changes reached statistical significance using the non-parametric Kolmogorov–Smirnov test (when comparing mean changes between the two groups) That significance was removed by the Bonferroni correction as the adjustment for multiple testing produced a rejection *p*-value of 0.0125. Effect sizes (Cohen’s *d*) ranged from small to medium, the largest was observed in Dynamic % (RMSSD). A positive effect means that the change of the >63 years group was larger than the change of the <43 years group.*

**FIGURE 4:**
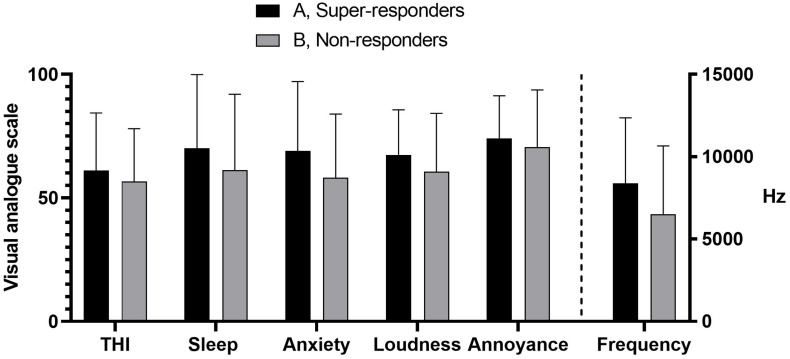
Comparison between taVNS responses and questionnaire-based clinical data and tinnitus frequency in groups A (super-responders) and B (non-responders), shown in Table 3. The mean values of THI, sleep disturbance, anxiety and tinnitus frequency were all greater in group A, but the differences were not statistically significant. Error bars represent standard deviations. THI = tinnitus handicap inventory.

To compare taVNS responses to questionnaire-based clinical data, we selected two patient groups, based on the magnitude of taVNS responses. Group A (21 patients) consisted of super-responders showing a post-taVNS RMSSD increase of 400–2000%. Group B (43 patients) consisted of non-responders showing unchanged or decreased post-taVNS RMSSD. Comparison of the values of THI, sleep disturbance and anxiety showed that group A comprized of more patients with mean THI scores > 60/100 and with sleep disturbance and anxiety scores > 60/100. However, the differences were not statistically significant (Table 3, Figure 4). Super-responders had also higher average tinnitus pitch (8.4 kHz) than non-responders (6.6 kHz) (Table 3, Figure 4).

**TABLE 3:** Comparison between taVNS responses and questionnaire-based clinical data in super-responders and non-responders.

	THI	Sleep	Anxiety	Loudness	Annoyance	Frequency
A. Super responders (RMSDD increase 400–2000%)	61,05 (*n* = 21)	70,00 (*n* = 20)	68,95 (*n* = 19)	67.35 (*n* = 19)	74.12 (*n* = 19)	8398 Hz (*n* = 20)
B. Non-responders (decrease or no change)	56,67 (*n* = 43)	61,22 (*n* = 37)	58,11 (*n* = 37)	60,63 (*n* = 37)	70,47 (*n* = 37)	6562 Hz (*n* = 40)
*p*-Values	0,246	0,642	0,198	0,147	0,197	0,122
Cohen’s *d*	0,301	0,128	0,358	0,429	0,386	0,414

*Comparison between taVNS responses and questionnaire-based clinical data and tinnitus frequency in two patient groups. Group A (*n* = 21): super-responders (taVNS-induced RMSSD increase of 400–2000%). Group B (*n* = 43): non-responders (RMSSD decreased or unchanged). The mean values of THI, sleep disturbance, anxiety and tinnitus frequency were all greater in group A, but the differences were not statistically significant (Student’s *t*-test). Bonferroni adjustment method for multiple testing produced a rejection *p*-value of 0.0083. Effect sizes (Cohen’s *d*) ranged from small to medium. A positive effect indicates that the mean of the super-responder group was larger than the mean of the non-responder group.*

### Results of VSEP Testing

There was a strong stimulation artifact (0 ms) after which oscillations were registered at about 3 ms. These have been earlier described to be of brainstem origin (VSEP) (Fallgatter et al., 2003). This response, however, was interpreted and presumed to be of local origin, arising from muscles in the ear region, not in the brainstem, in accordance to Leutzow et al. (2013). Therefore, we have not used VSEP as a biomarker for taVNS.

### Results of the 1-Year Follow-Up Outcome Questionnaires

**FIGURE 5:**
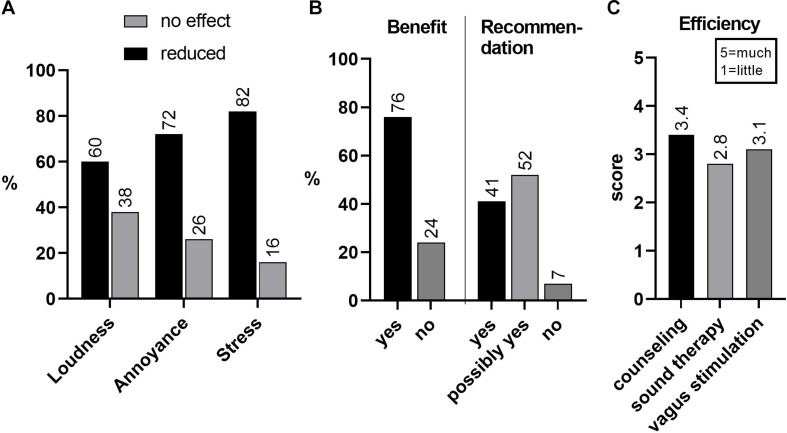
**(A)** Results of 1-year-follow-up outcome of 113 patients of which 78 (69%) data was received. Tinnitus annoyance had decreased in 72% and tinnitus-triggered stress in 82%. Symptoms had increased in 2%. **(B)** 76% of patients reported that they had benefited from TCPT treatment, including taVNS; 41% would recommend and 52% possibly recommend TCPT plus taVNS t for a friend or relative if suffering from similar health problems. **(C)** Of the components of treatment, counseling was reported most helpful (score 3.4 from the range 1–5), followed by taVNS (3.1) and sound therapy (2.8).

One-year follow-up therapeutic outcome data was possible to receive from 78 out of 113 patients (69%). Both the loudness and annoyance of tinnitus had decreased in about two thirds and, importantly, stress had decreased in more than 80% of patients (Figure 5A). About 76% of patients reported that they had benefited from the TCPT (including taVNS) treatment. When asked whether they would recommend similar treatment for a friend or near relative with similar health problems, 90% answered yes or probably yes (Figure 5B). When asked what constituent of the TCPT therapy regimen was most efficient in 1 to 5 scale, counseling showed an efficacy of 3.4, followed by taVNS (3.1) and sound therapy (2.8) (Figure 5C). Thus, it seems that taVNS is a useful addition to tinnitus treatments. We stress, however, that the most important constituent of tinnitus therapy is directive counseling. That has been our opinion for decades and it was also supported by the results of this follow-up study.

## Discussion

The main aim of this study is to share our experience of the usage of taVNS in the treatment of distressing tinnitus. Therefore, we discuss only matters that we feel important in clinical practice. Our study shows that taVNS is safe. Baseline HRV data of 171 patients showed that more than three quarters of TRMS patients had increased sympathetic prevalence (preponderance) before the first test-taVNS. The mean values of different HRV parameters changed toward increased parasympathetic activity by test-taVNS in about 80% of patients. These changes were more pronounced in patients showing greater tinnitus handicap, more severe associated symptoms, higher stress levels and higher age before the test stimulation. No significant adverse effects were reported in follow-up questionnaires. Our conclusion is that our tinnitus treatment program, including taVNS, alleviates stress and handicap caused by tinnitus.

The main problem of patients with disturbing moderate or severe tinnitus is usually TRMS (Andersson and Hesser, 2013) and associated imbalance of the ANS, leading to sympathetic prevalence and correspondingly reduced parasympathetic activity (Thayer et al., 2012; Chalmers et al., 2014). Therefore, the optimal TRMS treatment would be by trophotropic (parasympathetic activity enhancing) means. This could be achieved by behavioral methods such as cervical vagal massage, Valsalva maneuver or respiratory VNS (Gerritsen and Band, 2018) or by general relaxation generating methods such as yoga, mindfulness, biofeedback and cognitive behavioral therapy. The present study, in agreement with our previous results (Ylikoski et al., 2017), suggests that the therapeutic trophotropic effect can be accentuated by taVNS in TRMS and, thereby, tinnitus handicap can be attenuated. This would be in accordance with recent studies reporting that (implanted) VNS paired with tones as well as taVNS constitute promising novel treatments for tinnitus (Lehtimäki et al., 2013; Tyler et al., 2017; Yakunina et al., 2018). On the other hand, another clinical study did not report improvement of tinnitus with taVNS alone, although the therapy was found to be safe (Kreuzer et al., 2014).

The vagus nerve provides a unique therapeutical entrance to the CNS. Although VNS has become an established intervention therapy for therapy resistant epilepsy and depression, the exact mechanisms remain unsolved. Preclinical studies have shown that VNS therapy results in intermittently increased release of multiple neuromodulators, including norepinephrine, acetylcholine, serotonin and brain-derived neurotrophic factor (BDNF) (Hassert et al., 2004; Dorr and Debonnel, 2006). BDNF is a key player in the CNS neuronal plasticity. Maladaptive plasticity is thought to be involved in many medical entities, particularly in illnesses such as phantom pain, dystonias and tinnitus (Flor et al., 2001; Engineer et al., 2011; Kilgard, 2012). In addition, enhancement of neuronal plasticity through VNS has been shown to improve functional recovery in animal models and patients with stroke-induced upper limb paresis (Dawson et al., 2016; Engineer et al., 2019) and in animal models of spinal cord injury (Ganzer et al., 2018). BDNF, norepinephrine and serotonin have been suggested to play a key role in this enhanced plasticity (Hulsey et al., 2017). BDNF is an activity-dependent neurotrophic factor (Barde et al., 1982). Therefore, therapeutic sessions have consisted of VNS combined with simultaneous activities such as physical therapeutic movements in upper limb paresis or pairing with tones in tinnitus (Engineer et al., 2011; Dawson et al., 2016). In depression, the activity part is thought to consist of psychotherapy.

We emphasize that taVNS should not be applied as a solo but an adjunctive therapy. In our study the treatment of patients with distressing tinnitus was always started with a 1.5 h office visit during which patient‘s complaints were dealt with our TRT modification, the TCPT program. This consists of careful diagnostics with hearing and tinnitus profiling, counseling and instructions for sound therapy. The taVNS has been the fourth component of TCPT, initiated at the office visit and continued, together with sound therapy (the activity component), as home therapy. Our inquiries (Figure 5) indicate that counseling is the most important therapy constituent for this kind of distressed patients. During counseling, which takes about 1 h, we try to demystify tinnitus by explaining what tinnitus is all about and how one should behave in order to diminish its annoyance; not to be afraid that it worsens or never disappears. It has been shown that uncertainties and fears constitute a potent stressor and can easily cause diseases (Peters et al., 2017). Specifically, fear and anxiety can be significant co-factors, possibly modulated by amygdala, in triggering TRMS (Andersson et al., 1999; Cima et al., 2012). This type of counseling should be applied also to the therapeutic regimen of anxious and depressive patients.

### Mechanisms of TRMS

**FIGURE 6:**
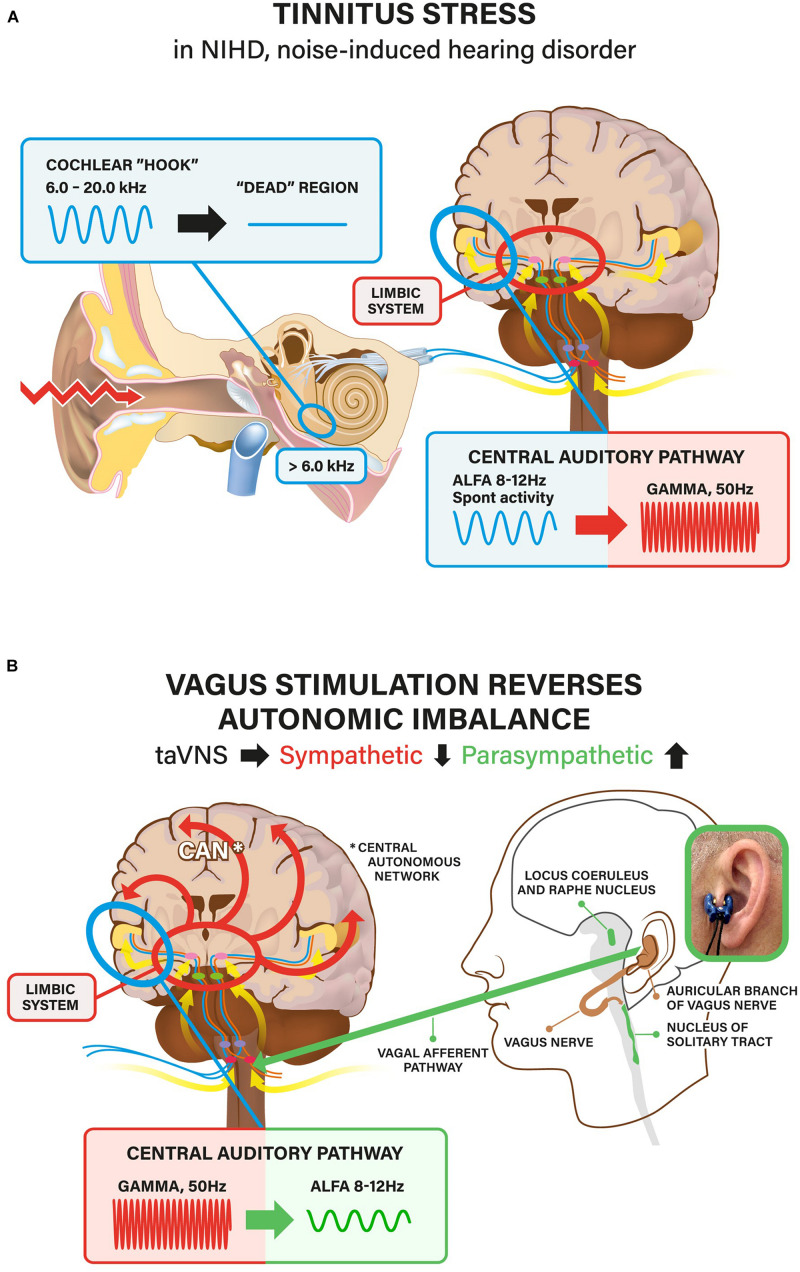
**(A)** Summary of the hypothesis. Noise exposure (most commonly music) causes dysfunction/damage in the high-frequency (>6.0 kHz) region of the cochlea) in >85% of patients. This leads to high-pitched tinnitus, most commonly at about 8.0 kHz. Bioelectrical impulse flow from the damaged cochlear region toward auditory cortex diminishes or ceases (”dead” region). This leads to reduced (inhibitory) regulation with subsequent neuronal hyperactivity in the central auditory pathway, first in the auditory nuclei of brainstem, later in the auditory cortex. The normal (spontaneous) alpha activity (in EEG) changes to gamma activity. The central auditory pathway is intimately connected to the limbic system (that controls emotions). Tinnitus is experienced as an emotionally negative sensation including uncertainties and fears (”what is this all about?”; “does it ever go away?”). Thereby, the perceptive (hearing) network is connected to the distress network (stress). The stressor leads to imbalance of the central autonomous network (CAN) with hyperactivity of the sympathetic nervous system (flight or fight or freeze response) and, correspondingly reduced activity of the parasympathetic nervous system (PNS) (relax, calm down). **(B)** Vagus nerve is the main player of the PNS. Therefore, activation of the vagal system increases PNS activity. For taVNS we have used a specially designed Salustim device that uses an ear-clip electrode inserted to the tragus and electrically stimulates ABVN. The taVNS reverses sympathetic hyperactivity in the limbic system and the CAN imbalance toward parasympathetic direction. Reduction of distress also facilitates the reversal of gamma-hyperactivity back to normal alpha-activity in the auditory central pathway.

The prominent feature of our patients was the SR (arousal) caused by tinnitus. SR is such a personal experience that it is not possible to ascertain – or even speculate- why most of the patients had developed a severe SR. Also, the reliability of our main markers for SR, the questionnaires and HRV tests, are not accurate and often even debatable. However, there are some general clinical and test-based characteristics, which allow to suggest speculative stress pathways at least in the patients in which tinnitus was initially triggered by exposure to excessively loud sounds. These patients had contracted NIHD that is now known to be the most common consequence of noise trauma. Its most common symptoms are tinnitus and hyperacusis, the audiometrically measurable hearing impairment being a much less common feature (Kähäri et al., 2001; Szibor et al., 2018). Usually, the intensity of the exposing sound (mostly music) is relatively low in NIHD and its action on the cochlea is thought to be cellular stress/damage that does not lead to significant hair cell loss. Low-level noise exposure is known to cause synaptopathy, the damage of the synapses between inner hair cells and auditory nerve fibers (Kujawa and Liberman, 2009). It is generally accepted that tinnitus frequency and the predicted location of the lesion along the tonotopic axis of the cochlear are closely related. In the present study, tinnitus frequency tended to be very high, higher than 6 kHz in more than 85% of cases, indicating that the presumable cellular stress response was localized to the extreme basal coil of the cochlea. Most researchers agree that tinnitus can be linked to changes at one or more relays along the peripheral and central auditory pathways including auditory cortex (Jastreboff, 1990; Lockwood et al., 1998; Giraud et al., 1999; Møller, 2003; Eggermont and Roberts, 2004; Rauschecker et al., 2010). Although it is generally accepted that the dysfunction of the auditory system is necessary for tinnitus to occur, it is unclear whether this defect alone is sufficient to cause chronic tinnitus or whether additional mechanisms outside the auditory-sensory regions are involved. Clinically, there is a clear relationship between tinnitus and the emotional state (Sullivan et al., 1988; Dobie, 2003) and it has led to the suggestion that the limbic system plays a role in modulating or perpetuating tinnitus (Jastreboff, 1990; Rauschecker et al., 2010). Indeed, the lifetime incidence of clinical depression in tinnitus patients is estimated to be more than twice of the national average (∼35% vs. 15%, respectively, Folmer et al., 1999). Treatment regimens that include forms of cognitive-behavioral therapy have been shown to be effective for many tinnitus patients (Jastreboff, 2007; Robinson et al., 2008). Although the exact nature of the involvement of the limbic system in chronic tinnitus remains to be shown, there is substantial – mainly neuroimaging – evidence indicating that networks such as the corticostriatal circuit and the amygdala-anterior cingulate cortex axis are involved (Leaver et al., 2011; Chen et al., 2017; Xu et al., 2019). It has been shown that the corticostriatal circuit, which includes the nucleus accumbens and ventromedial posterior frontal cortex, does indeed differ in the brains of individuals with tinnitus (Leaver et al., 2011). The corticostriatal circuit is part of the general “appraisal network”, determining which sensations are important and ultimately affecting how (or whether) those sensations are experienced (Simmons et al., 2020). Our theory of the pathogenetic mechanism of NIHD is schematically summarized in Figure 6.

### The Inflammatory Reflex or Neuroinflammation in the Pathogenesis of Stress and Tinnitus, and Possible Attenuation by taVNS

Although the common pathways between stress exposure and pathophysiological processes leading to tissue damage are still debatable, several results indicate that stress can activate an inflammatory response in the brain and in the periphery (Calcia et al., 2016, for review). In this damaging process, stress-induced pro-inflammatory factors including C-reactive protein, IL-6, TNFα, IL-1β and NF-κB, have an important role (Miller et al., 2008). In common, over-activated immune system, increased sympathetic nervous system activity and reduced glucocorticoid (GC) responsiveness may work tandemly in the activation of inflammatory responses during stress. GCs, catecholamines, cytokines and other mediators are thought to be the main mediators of the stress-induced pro-inflammatory effect. Correspondingly, when the auditory system in a rodent model was exposed to acoustic overstimulation causing hearing impairment and tinnitus, neuroinflammation in the central auditory system was found to be importantly involved (Wang et al., 2019).

After exposure to acoustic overstimulation from loud noise or music, the resulting tinnitus is high-pitched (Szibor et al., 2018). It can be very difficult to tolerate and habituate this tinnitus and, therefore, it may lead to sleep disturbances, anxiety and finally to SR. Tinnitus can be regarded as the consequence of multisensory interactions between the auditory and limbic systems. This is because extensive functional networks and tinnitus distress strongly correlate with enhanced effective connectivity that is directed from the amygdala to the auditory cortex (Rauschecker et al., 2010; Chen et al., 2017). When the stimulation patterns and dynamics of functional networks during VNS were examined by fMRI, the vagus nerve was found to convey signals to the brain through the polysynaptic neuronal pathways, by projecting to the brainstem nuclei (NTS, locus coeruleus), subcortical areas and lastly to the cortex (Henry, 2002; Ressler and Mayberg, 2007), thus covering the entire CAN. fMRI and a spatially independent component analysis were utilized in a recent experimental study (Cao et al., 2017). That study demonstrated that VNS activated 15 out of 20 brain networks and that the activated regions covered > 75% of the brain volume.

Very soon after the acoustic trauma, which means during ongoing inflammatory response or neuroinflammation, patients usually seek medical assistance because of uncertainty (and fear) with questions regarding possible consequences and management. If no appropriate treatment or even counseling are available and only negative counseling is offered (“nothing can be done”), a complete SR with self-perpetuating cycle develops: distress worsens tinnitus and worsening tinnitus accentuates SR. This is about the clinical picture characterizing most of the patients included in the present study. Therefore, it is not surprising that our TCPT therapeutic regimen, including taVNS as the adjunctive treatment, significantly benefited the great majority of patients. In this type of condition, taVNS may be especially effective, perhaps due to a dual action: it may attenuate the underlying neuroinflammation or inflammatory process in parallel or subsequent to SR.

Of special interest are our findings indicating that aged patients are more responsive to acute taVNS than younger ones, as revealed by HRV tests. Our results are preliminary and appropriate controls are missing, but if age-related differences in HRV responses hold true also in controlled studies, it may open new avenues for the treatment of hearing disorders, particularly the two most common disorders, presbyacusis and NIHD. There is no effective treatment available for them today. Targeting neuroinflammation with taVNS might be a novel therapeutic possibility for NIHD with tinnitus. There is strong preclinical scientific evidence of the beneficial role of VNS in the treatment of immunologic reflex-associated disorders, particularly rheumatoid arthritis [reviewed by Tracey (2018)]. As a method taVNS is safer than VNS, because ABVN has no efferent neurons.

In the pathogenesis of AF, another common medical entity, accumulating evidence indicates that the inflammatory pathways play a significant role (Aviles et al., 2003; Hu et al., 2015). In a recent clinical trial, chronic, intermittent taVNS (with the Salustim device used in this study as well) resulted in lower AF burden in about 50% of patients compared to sham control stimulation. These results support the use of taVNS to treat paroxysmal AF in selected patients (Stavrakis et al., 2020).

### How to Improve the Efficacy of taVNS?

The stimulation of ABVN is an easy and non-invasive method to obtain the beneficial effects of vagal system activation. However, there are still uncertainties concerning the modes of stimulation, including the optimal stimulation site and parameters. These can be defined only after the appropriate biomonitoring tests become available. While clinical taVNS applications have been widely noted in the literature, the physiological mechanisms supporting such clinical effects are poorly understood, particularly in humans.

May be the most important proof of the usefulness of taVNS has so far been obtained from clinical studies. taVNS has been employed for patients suffering from various disorders, including epilepsy (Stefan et al., 2012), tinnitus (Lehtimäki et al., 2013; Yakunina et al., 2018), depression (Rong et al., 2012; Hein et al., 2013), pain (Napadow et al., 2012; Laqua et al., 2014; Janner et al., 2018) and migraine (Straube et al., 2015; Garcia et al., 2017). Clinical studies do not, however, directly show that the beneficial effects are due to ABVN stimulation. This is because the outer ear has an innervation not only from the cranial nerve X, but also from the cranial nerves VII and V as well as the cervical plexus.

Much of our present understanding of the mechanisms and presumed efficacy of taVNS comes from fMRI studies. These studies have shown that taVNS produces significant cortical effects in the vagal afferent pathway. Thus, outer ear stimulation in the regions innervated by ABVN activates afferent vagal networks (Kraus et al., 2007; Dietrich et al., 2008; Frangos et al., 2015; Yakunina et al., 2017; Badran et al., 2018a). These studies have, however, failed to convincingly demonstrate that taVNS activates the crucial brainstem nuclei such as NTS. This has now changed when it was recently demonstrated using a ultrahigh-field (7T) fMRI that taVNS evokes activation in the ipsilateral NTS and upstream monoaminergic source nuclei of the brainstem (Sclocco et al., 2019). This finding supports the idea that the selective stimulation of ABVN is responsible for NTS activation. Corresponding selective NTS activation, comparable to tragal ABVN stimulation, may be possible by using percutaneous ABVN stimulation (Kaniusas et al., 2019). Percutaneous stimulation must, however, be considered as a mini-invasive procedure, because the skin is penetrated. It may be appropriate for the treatment of diseases in medical offices, but not for continuous home-therapy.

Importantly, NTS activity is known to be modulated by respiration, both through the bottom-up afferent pathway from pulmonary stretch receptors and aortic baroreceptors and through the top-down effects from respiratory nuclei in the medulla (Sclocco et al., 2019). Specifically, NTS receives inhibitory influence during inhalation and facilitatory influence during exhalation (Miyazaki et al., 1999; Baekey et al., 2010). Therefore, it has been proposed that NTS targeted by taVNS can be enhanced by gating stimulation to the exhalation phase of the respiratory cycle via respiration-gated auricular vagal afferent nerve stimulation (RAVANS) (Napadow et al., 2012; Garcia et al., 2017). Our taVNS treatment protocol includes instructions for slow breathing (“Respiratory VNS”) (Gerritsen and Band, 2018), but electrical stimulation in our device was not synchronized to give electrical stimuli specifically during exhalation.

### HRV

Because HRV is a biomonitor for ANS function, we also analyzed HRV changes before and immediately after acute test-taVNS. HRV as well as HR have been found to be useful in monitoring the effects of taVNS (Clancy et al., 2014; Antonino et al., 2017; De Couck et al., 2017; Ylikoski et al., 2017; Badran et al., 2018b; Bretherton et al., 2019). Clancy et al. (2014) demonstrated that 15 min of taVNS administered to the tragus significantly increased HRV, at least partly through reduction of sympathetic nerve activity. In addition, the acute taVNS has been demonstrated to improve spontaneous baroreflex sensitivity (BRS) that may be the most sensitive measure of ANS function and thereby the parasympathetic activity (Antonino et al., 2017; Bretherton et al., 2019). Normal aging is associated with increase in sympathetic prevalence and/or decreases in the vagal tone and overall variability, which is reflected in HRV (Stein et al., 1997; Kuo et al., 1999). There is a general consensus that we all have our own dominant parasympathetic and sympathetic regulation that gradually decreases with advancing age due to a significant reduction of nocturnal parasympathetic activity. Hence, the preservation of parasympathetic function may serve as a biomarker related to the healthy longevity and vitality in late life span (Zulfiqar et al., 2010). In addition to normal aging, a shift toward sympathetic prevalence may contribute to age-related conditions, such as hypertension, heart failure and AF. Evidence suggests that taVNS could play a role in ameliorating these conditions. Bretherton et al. (2019) have suggested that age-related autonomic dysfunction (decrease of HRV and BRS), QoL, mood and sleep changes improve with taVNS administered daily for 2 weeks. This is in line with our previous study (Ylikoski et al., 2017) and also with the observations in the present study showing that the HRV improvement after acute test-taVNS was greater in elderly individuals (with TRMS) than in younger ones. The findings of Bretherton et al. (2019) also point to the influence of initial values in determining the magnitude and direction of change following taVNS: high initial sympathetic prevalence, tension, anger, depression as well as low energy and sleep quality were associated with greater improvements of HRV and BRS. This is also in line with our findings of HRV changes after acute test-taVNS: when the HRV-RMSSD values were correlated to clinical data, patients with high scores in THI, tinnitus annoyance, sleep disturbances and anxiety showed largest changes in RMSSD. Overall, our findings support the idea of Bretherton et al. (2019), when they state: “considering the ease of application and affordability of taVNS, there is significant potential in attenuating symptoms associated with age-related conditions and prolonging the period of healthy ageing.”

### VSEP

Different physiological and neurophysiological tests have been used to biomonitor the effects of taVNS. According to the literature, VSEP is the most useful online biomonitor. Therefore, we investigated whether VSEP could be used for our biomonitoring purposes. In order to reveal the anatomic site where VSEP arises (hypothetically NTS of the brainstem), we registered VSEP responses using EGI GTEN 100 EEG system with 256 electrodes (Palva and Palva, 2018) and with multiple stimulation parameters. We found a strong stimulation artifact (0 ms) and thereafter oscillations at about 3 ms, previously described to originate from the brainstem. We interpreted that this response has a local origin, presumably arising from muscles in the ear region, not in the brainstem, in accordance to Leutzow et al. (2013). Therefore, we had to abandon VSEP as a biomarker for taVNS. VSEP seemed to be unrelevant also because of the low numbers (50–100) of epochs reported in prior VSEP studies (Fallgatter et al., 2003; Polak et al., 2007). It is well known that in the most commonly employed brainstem response test, auditory brainstem response (ABR), the minimal number of epochs needed for reliable results vary between 500 and 1000. We are currently investigating whether other features of EEG could be used as online biomonitoring methods for taVNS.

### Limitations

The results of the present study should be interpreted with caution because they only represent a retrospective clinical cohort study. However, all our clinical data are based on structured diagnostic forms and questionnaires that were used in the management of the patient population. Furthermore, the consistent improvement of HRV -a seemingly useful marker for mental stress- in 80–90% of our patients suggests that taVNS is a useful (adjunctive) therapeutic means in severe tinnitus. This study encourages future controlled clinical studies on the usefulness of taVNS in tinnitus. The major defect in our retrospective study is the lack of appropriate controls and sham procedures, which are the crucial components of prospective randomized controlled trials (RCTs). Our patient population consists of nonselective series in contrast to that of RTCs in which participants are recruited through various procedures enhancing the selection factor. This aspect is particularly important when the target of investigation is such a common symptom as mental stress.

## Conclusion

TRMS is an example of a tinnitus-triggered stress condition in which patients may benefit from taVNS. Our clinical data and HRV results before and after test-taVNS suggest that patients with TRMS have ANS imbalance with increased sympathetic activity and, correspondingly, reduced parasympathetic function. Acute test-taVNS increased parasympathetic activity, more in elderly and patients with more severe stress symptoms. Although our follow-up outcome study primarily aimed to study the TCPT therapeutic efficacy in patients with TRMS, showing that this therapeutic program alleviated tinnitus severity, the results can also be interpreted such that the majority of stressed tinnitus patients get additional benefit from taVNS as an adjunct therapy. HRV seems to serve as an easy and rapid method for assessment of SR and thereby ANS balance. Combining clinical data to HRV results may be useful in selecting patients for taVNS. We have now clinical experience on the long-term use of taVNS by several of our patients. They have used taVNS daily for more than 4 years without any adverse effects. They continue to use the device because of subjective benefits. Currently, at the same setting where the present study was performed, we offer taVNS treatment for all our patients who show THI scores of 34 or over. We regard this as an alternative to a possible need for e.g., tranquilizers. However, taVNS should not be used as a solo therapy but as an adjunct to a treatment program in which all the constituents are aimed to restore the sympathovagal imbalance through parasympathetic activation. Generally, this study offers additional support to the idea that taVNS might offer a new, targeted therapeutic tool for patients in whom sympathovagal imbalance is involved. Furthermore, taVNS is patient-friendly and of low-cost. However, as there are not (yet) appropriate online biomarkers available for taVNS, there is still a great need for additional research to find optimal therapeutic regimen as well as better stimulating devices.

## Data Availability Statement

The raw data supporting the conclusions of this article will be made available by the authors, without undue reservation.

## Ethics Statement

Ethical review and approval was not required for the study on human participants in accordance with the local legislation and institutional requirements. Written informed consent from the participants’ legal guardian/next of kin was not required to participate in this study in accordance with the national legislation and the institutional requirements.

## Author Contributions

JY contributed to the design of the study, interpretation of data, and writing the manuscript. JY, JL, and MY conducted the clinical work. MM conducted the data analysis and illustrations. UP, ZJ, SS, and AM revised the manuscript. All authors contributed to the article and approved the submitted version.

## Conflict of Interest

JY, JL, and MY are board members of the Helsinki Ear Institute and Salustim Group. The remaining authors declare that the research was conducted in the absence of any commercial or financial relationships that could be construed as a potential conflict of interest.

## Abbreviations

ABVN: auricular branch of the vagus nerve
AF: atrial fibrillation
ANS: autonomous nervous system
CAN: central autonomic network
CNS: central nervous system
ECG: electrocardiogram
HR: heart rate
HRV: heart rate variability
MIT: music-induced tinnitus
NIHD: noise-induced hearing disorder
NIT: noise-induced tinnitus
NTS: nucleus tractus solitarius
SR: stress reaction
taVNS: transcutaneous VNS
TCPT: tinnitus care pathway technology
THI: tinnitus handicap inventory
TRMS: tinnitus-related mental stress
TRT: tinnitus retraining therapy
VAS: visual analog scale
VNS: vagal nerve stimulation
VSEP: vagal somatosensory evoked potential.
